# Evaluation of Monocarboxylate Transporter 4 (*MCT4)* Expression and Its Prognostic Significance in Circulating Tumor Cells From Patients With Early Stage Non-Small-Cell Lung Cancer

**DOI:** 10.3389/fcell.2021.641978

**Published:** 2021-04-22

**Authors:** Athina Markou, E. Tzanikou, G. Kallergi, E. Pantazaka, V. Georgoulias, A. Kotsakis, E. Lianidou

**Affiliations:** ^1^Analysis of Circulating Tumor Cells, Lab of Analytical Chemistry, Department of Chemistry, University of Athens, Athens, Greece; ^2^Division of Genetics, Cell and Developmental Biology, Department of Biology, University of Patras, Patras, Greece; ^3^First Department of Medical Oncology, IASO General Hospital of Athens, Athens, Greece; ^4^Department of Medical Oncology, University General Hospital of Larissa, Thessaly, Greece

**Keywords:** liquid biopsy, CTCs, NSCLC, *MCT4*, RT-qPCR, EMT 3

## Abstract

**Purpose:** Monocarboxylate transporter 4 (*MCT4)* can influence the amount of lactate in the tumor microenvironment and further control cancer cell proliferation, migration, and angiogenesis. We investigated for the first time the expression of *MCT4* in circulating tumor cells (CTCs) derived from early stage Non-Small Cell Lung Cancer patients (NSCLC) and whether this is associated with clinical outcome.

**Experimental Design:** A highly sensitive RT-qPCR assay for quantification of *MCT4* transcripts was developed and validated and applied to study *MCT4* expression in CTC isolated through the Parsortix size-dependent microfluidic device from 53 and 9 peripheral blood (PB) samples of NSCLC patients at baseline (pre-surgery) and at relapse, respectively, as well as the “background noise” was evaluated using peripheral blood samples from 10 healthy donors (HD) in exactly the same way as patients.

**Results:** *MCT4* was differentially expressed between HD and NSCLC patients. Overexpression of *MCT4* was detected in 14/53 (26.4%) and 3/9 (33.3%) patients at baseline and at progression disease (PD), respectively. The expression levels of *MCT4* was found to increase in CTCs at the time of relapse. Kaplan-Meier analysis showed that the overexpression of *MCT4* was significantly (*P* = 0.045) associated with progression-free survival (median: 12.5 months, range 5–31 months).

**Conclusion:** *MCT4* overexpression was observed at a high frequency in CTCs from early NSCLC patients supporting its role in metastatic process. *MCT4* investigated as clinically relevant tumor biomarker characterizing tumor aggressiveness and its potential value as target for cancer therapy. We are totally convinced that *MCT4* overexpression in CTCs merits further evaluation as a non-invasive circulating tumor biomarker in a large and well-defined cohort of patients with NSCLC.

## Introduction

Lung cancer remains the most commonly diagnosed cancer and the leading cause of cancer death globally. Non-Small Cell Lung Cancer (NSCLC) is the common histological subtype of the disease, accounting for 85% of all lung cancer diagnoses (Molina et al., 2008). Almost 45% of patients with operable early stage NSCLC relapse within the first 18 months and the probability of patients’ survival depends on the possibility of early detection of relapse (Siegel et al., 2015). The use of targeted therapy such as tyrosine kinase inhibitors (TKIs) and/or immunotherapy has led to unprecedented survival benefits in selected patients (Wu et al., 2020). Moreover, the use of therapeutic modalities against the Minimal Residual Disease (MRD) and before the development of clinically detectable metastatic lesions seems to be emerged as an important advancement in the management of early stage NSCLC. Indeed, it has been recently reported that the administration of Osimertinib in the context of adjuvant treatment in resected EGFR mutant NSCLC significantly reduced the relapse rate and prolonged the overall survival (Wu et al., 2020). The possibility of probing the early detection of NSCLC via a blood draw –termed as “liquid biopsy”- has attracted remarkable interest among the oncology community (Pawlikowska et al., 2019; Tamminga et al., 2019; Frick et al., 2020). Different tumor-derived components can be isolated from blood, including Circulating Tumor Cells (CTCs), circulating tumor DNA (ctDNA), cellfree RNA (cfRNA), exosomes, and tumor-educated platelets (TEP), providing information about the dynamic tumor profile over time (Heidrich et al., 2020). The FDA has approved the use of ctDNA for the response prediction and monitoring development resistance to EGFR TKI therapy in NSCLC patients (Wang et al., 2017; Zhang et al., 2017).

The application of liquid biopsy in NSCLC is being used for the diagnosis, prognosis and monitoring of disease based on signature molecular markers (Luo et al., 2018). However, the clinical significance of CTC enumeration in NSCLC is yet to be established since due to the EMT process, EpCAM-independent methods are required in order to isolate and characterize CTCs from NSCLC patients. Indeed, molecular characterization of CTCs got potential for the improvement of our knowledge in the field of metastatic process, the identification of new treatment predictive markers and stratification of patients into prognostic groups.

The CellSearch® system, which is the only FDA approved assay for CTC detection in metastatic breast, colorectal and prostate cancers but is able to identify CTCs only in about 23–39% of stage IV NSCLC patients (Krebs et al., 2012). Detection of CTCs in NSCLC has been challenging due to the rarity of these cells in circulation and the presence of non-epithelial characteristics due to epithelial mesenchymal transition (EMT) (Lianidou et al., 2015). EMT is characterized by down-regulation of epithelial markers, such as cytokeratin’s (CKs), and up-regulation of mesenchymal markers like *Vimentin* (*VIM*).

Three metabolic properties of cancer cells (a) glucose uptake, (b) lactate secretion and (c) oxygen availability constitute the Warburg effect which attracts the interest of the scientific community for a many years (Warburg, 1956; DeBerardinis and Chandel, 2020). Moreover, Reprogramming Energy Metabolism is an Emerging Hallmark for cancer (Hanahan and Weinberg, 2011). Monocarboxylic acids including lactate play a crucial role in cellular metabolism, and their regulation has become a new target for understanding the pathogenesis of abnormal cellular processes such as oncogenesis (Poole and Halestrap, 1993; Halestrap and Price, 1999). These acids must be rapidly transported across the plasma membrane of cells and this transportation is mediated by proton-linked monocarboxylate transporters (MCTs). *MCT4* is highly expressed in glycolytic tissues such as white skeletal muscle fibers, astrocytes, white blood cells, and chondrocytes, and it plays an important role in lactate efflux from cells (Meredith and Christian, 2008). *MCT4* can control the amount of lactate in the tumor microenvironment regulating cancer cell proliferation, migration, and angiogenesis. *MCT4* expression in the tumor microenvironment has been associated with decreased overall survival (OS) (Nakayama et al., 2012; Baek et al., 2014; Doyen et al., 2014; Zhu et al., 2014), and decreased disease-free survival (DFS) in cancer patients (Curry et al., 2013; Doyen et al., 2014; Zhu et al., 2014). Moreover, *MCT4* has been proposed as a new therapeutic target in several tumor types including NSCLC (Kim et al., 2018; Kuo et al., 2020; Puri and Juvale, 2020).

In the current study, we first developed and validated a highly sensitive RT-qPCR assay for the quantification of *MCT4* transcripts, and report for the first time that *MCT4* is overexpressed in CTC isolated from patients with early NSCLC. We further evaluated whether *MCT4* overexpression in CTC is associated with DFS. Our findings indicate that *MCT4* overexpression in CTCs should be prospectively evaluated as a potential biomarker for early relapse in patients with resected NSCLC.

## Materials and Methods

### Clinical Samples

Fifty three patients with early stage NSCLC were enrolled in the study and 62 peripheral blood samples (25 mL in EDTA tubes) from these patients were prospectively collected; 53 samples were obtained at baseline (pre-surgery), and 9 samples at the time of relapse whilst 10 peripheral blood samples from healthy donors (HD) were used as controls. For 12 patients that have been randomly chosen, 10 mL peripheral blood from the same blood draws were used to perform CTCs’ IF analysis. The first 5 mL of blood were discarded in order, to avoid contamination from skin epithelial cells. All patients gave a written informed consent to participate in the study, which was approved by the Ethics and Scientific Committee of Thoracic Diseases General Hospital Sotiria. All HD had no known illness or fever at the time of draw, no history of malignant disease, and were ≥35 years old. Clinical samples were collected from 32 men and 21 women (median age: 65.2 years, range: 39–81) and all patients were diagnosed with operable (stage IA–IIIA) NSCLC. 23 patients were diagnosed with adenocarcinoma (ADC), 26 with Squamous Cell Carcinoma (SCC) and 4 with undifferentiated (NOS) NSCLC. Thirty-eight (71.6%) patients had no evidence of disease infiltration in resected lymph nodes (N0 disease). The main patients’ characteristics are summarized in Supplementary Table 1.

### CTCs Enrichment Using the Parsortix Size-Based Microfluidic Device

Micro-fluidic device named as Parsortix (ANGLE plc, United Kingdom) (Hvichia et al., 2016) was used to for the isolation of CTCs from 25 mL whole blood. A microscope slide sized disposable cassette was used for the division of blood components (Chudziak et al., 2016; Porras et al., 2018). After that, CTCs were collected in a total volume of 200 μL of PBS into tubes. The isolation of total RNA from enriched CTCs was performed by TRIZOL-LS (Thermo Fisher Scientific, United States), and finally cDNA synthesis of the extracted total RNA was carried out as previously described (Strati et al., 2017; Zavridou et al., 2018).

### CTCs Isolation by ISET System

For the isolation of CTCs with the ISET (Isolation by SizE of Tumor cells) platform (Rarecells Diagnostics, France) 10 mL of peripheral blood was used. At first, each sample was diluted in 1:10 ISET buffer (Rarecells Diagnostics) and was incubated for 10 min at room temperature (RT). 100 mL of the diluted sample was filtered using a depression tab adjusted at −10 kPa. Finally, the membrane was dried for 2 h at RT and stored at −20°C. Each membrane spot was used for identification of CTCs after immunostaining and fluorescence microscopy analysis (Kallergi et al., 2016).

### RT-qPCR Assay for *MCT4* Expression

*In silico* study for the design of the primers and TaqMan probes for *MCT4* and *B2M* (used as a reference gene) was carefully performed using Primer Premier 5.0 software. In order to ensure the specificity of all primers and probe sequences BLAST analysis was carried out (NCBI, nucleotide BLAST). Moreover, we carefully designed our primers and probes to completely avoid primer–dimer formation, false priming sites, formation of hairpin structures and hybridization to genomic DNA. The sequences of primers and probes are available in Supplementary Table 3.

RT-qPCR was performed in the LightCycler^®^ 480 instrument (Roche, Germany). Detailed optimization experiments were carried out (results not shown). The amplification reaction mixture for *MCT4* contained 2 μL of the PCR synthesis buffer (5×), 1 μL MgCl_2_ (25 mM), 0.2 μL dNTPs (10 mM), 0.15 μL BSA (10 μg/μL), 0.1 μL Hot-Start DNA polymerase (Promega), 0.3 μL of forward and reverse primer (10 μM), 1 μL hydrolysis probe (3 μM) and H_2_O to a final volume of 10 μL while the amplification reaction mixture for B2M contained 1 μL of PCR synthesis buffer (5×), 1.2 μL MgCl_2_ (25 mM), 0.15 μL dNTPs (10 mM), 0.3 μL BSA (10 μg/μL), 0.1 μL Hot-Start DNA polymerase (Promega), 0.25 μL of forward and reverse primer (10 μM), 0.83 μL hydrolysis probe (3 μM) and H_2_O to a final volume of 10 μL. Each experimental procedure included one positive and one negative control. cDNA from MCF-7 cell line was used as a positive control. In order to ensure that amplification of gDNA was completely avoided, four genomic DNAs at high concentrations were used as templates. None of these DNA samples were amplified. *B2M* was used as a reference gene for RT-qPCR. I n addition, single RT-qPCR was performed for epithelial markers (*CK-19*, *CK-8*, *CK-18*) and for EMT markers (*TWIST-1* and *VIM*) as previously described (Markou et al., 2018; Strati et al., 2019).

RT-qPCR data for *MCT4* expression were normalized in respect to *B2M* expression in the same cDNAs, using the 2^–ΔΔ*Ct*^ approach (Livak and Schmittgen, 2001). CTCs isolated through micro-fluidics device are not 100% pure; since the presence of co-isolated PBMC in CTC fractions could affect the specificity of the MCT4 assay, we evaluated this “background noise” by analyzing peripheral blood samples from 10 HD in exactly the same way as patients. We estimated a cut-off based on *MCT4* normalized expression in respect to *B2M* expression in this control group. Using this approach we defined a sample as positive for *MCT4* overexpression (MCT4 positive) based on the fold change of *MCT4* expression in the CTC fraction in respect to the corresponding fraction in the group of the 10 HD.

### Immunostaining and Confocal Imaging

ISET filters were washed with PBS and permeabilized with 0.5% Triton-X-100 in PBS for 10 min at 20°C. Non-specific antibody binding was blocked by incubation with 10% FBS in PBS for 1 h at 20°C. To identify CK, filters were incubated with a cocktail of mouse anti-CK7 antibody (Invitrogen, United States; 1:100 dilution in PBS with 1% FBS) and mouse A45 B/B3 anti-human cytokeratin (Amgen, United States); 1:70 dilution in PBS with 1% FBS) for 1 h at 20°C. Filters were washed (3 × 5 min) with PBS and then incubated with goat anti-mouse Alexa Fluor^®^ 488 secondary antibody (Life Technologies, United States; 1:500 dilution in PBS with 1% FBS) for 45 min at 20°C. Filters were washed (3 × 5 min) with PBS and then incubated with rabbit anti-VIM antibody (Abcam, 1:500 dilution in PBS with 1% FBS) for 1 h at 20°C. After washing (3 × 5 min) with PBS, filters were incubated with goat anti-rabbit Alexa Fluor^®^ 555 secondary antibody (Life Technologies; 1:600 dilution in PBS with 1% FBS) for 45 min at 20°C. Filters were washed (3 × 5 min) with PBS and mounted in Prolong anti-fade mounting medium containing DAPI (Cell signaling, United States). Slides were stored at −20°C before confocal imaging.

Cytospins with H 1299 cells were used as controls (Supplementary Figure 1). One positive control (stained with all antibodies) and two negative controls (omitting one of the first antibodies) were used. Slides were washed with PBS and cells were fixed/permeabilized with ice-cold acetone/methanol (9:1) for 20 min and, thereafter, non-specific antibody binding was blocked by incubation with 5% FBS overnight at 4°C.

All imaging used a Leica (Germany) TCS SP8 confocal microscope with a ×40 oil-immersion objective. In all dual-labeling analyses we confirmed, with the use of the controls, that there was no bleed-through between the two wavelengths. Cyto-morphological criteria proposed by Meng et al. (2004) (such as high nuclear/cytoplasmic ratio, etc.) were used in order to characterize a CK-positive cell as a CTC.

### Statistical Analysis

SPSS program was used for the statistical analysis of our data (SPSS Statistics 25.0, company, Armonk, NY, United States). In order to estimate the differences between groups chi-square test of independence or Fisher exact test (SPSS, version 25.0) was used. Kaplan–Meier analysis used to evaluate “time-to-event” data. Parametric and non-parametric tests were used to compare continuous variables between groups. Non-parametric tests were used to analyze the relationship between *MCT4* expression and various clinicopathological characteristics for each patient (the Mann–Whitney and χ^2^-test between 2 groups and the Kruskall Wallis test for 3 or more groups). All *P*-values are two-sided. A level of *P* < 0.05 is considered statistically significant unless specified otherwise.

## Results

**FIGURE 1:**
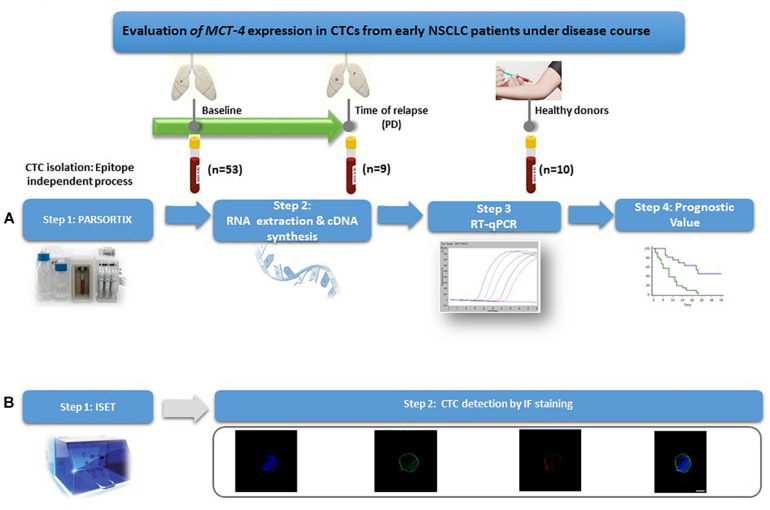
Outline of the experimental procedure. **(A)** Gene expression profiling of CTCs. **(B)** Phenotypic evaluation of CTCs.

The outline of the study is shown in Figure 1.

### Phenotypic Evaluation of *CK* and *VIM* in CTCs

CTCs were detected in 14 of 16 (87.5%) patients with early stage NSCLC. The mean and median numbers of CTCs per patient were 4.6 and 1 (range, 0–20), respectively. The absolute number of CTCs per patient for each distinct phenotype is shown in Table 1. Double-staining experiments (*VIM/CK*) and confocal laser scanning analysis revealed an heterogeneous expression of *CK* and *VIM.* Indeed, CTCs had low expression of *CKs* (lower than the CK expression in control H 1299 cells) (Supplementary Figure 2); *VIM*^+^*CK*^*low*^ CTCs could be detected in 12 out of 16 patients (75%) and *VIM^–^CK*^*low*^CTCs in 37.5% (6 of 16) of patients.

**TABLE 1:** The absolute number of CTCs per patient for CK^*low*^VIM^+^ and CK^*low*^VIM^–^ phenotype.

	**Number of cells**	
**PATIENT**	**CK^*low*^VIM^+^**	**CK^*low*^VIM^–^**
#1	8	6
#2	5	0
#3	5	2
#4	4	0
#5	3	0
#6	20	0
#7	0	2
#8	3	0
#9	11	1
#10	6	0
#11	3	0
#12	0	0
#13	1	0
#14	3	1
#15	0	0
#16	0	3

### Gene Expression of CTCs Using RT-qPCR

#### Epithelial Markers

**FIGURE 2:**
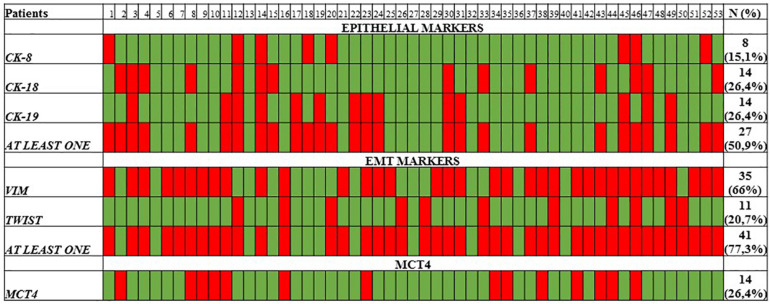
Heat map of *CK-8/CK-18/CK-19* epithelial markers, *VIM*, *TWIST-1*, and *MCT4* as quantified by RT-qPCR.

cDNAs isolated from clinical samples were further analyzed for epithelial markers (*CK8, CK18, CK19*) using our previously developed and analytically validated RT-qPCR assays. At baseline, *CK8* expression was detected in 8/53 (15.1%), while *CK18* and *CK19* were detected in 14/53 (26.4%) and 14/53 (26.4%), respectively. In total, in 27/53 (50.9%) samples were detected at least one CK marker (Figure 2).

#### EMT Markers

The expression of EMT markers *TWIST-1* and *VIM* was also evaluated in the same cDNAs, using our previously developed and analytically validated RT-qPCR assay (Markou et al., 2018; Strati et al., 2019). *TWIST-1* was overexpressed in 11/53 (20.7%) of samples, while *VIM* in 35/53 (66%) of samples. In total, at least one EMT marker was detected in 41/53 (77.3%) samples (Figure 2).

### *MCT4* Expression in CTC of Healthy Individuals and NSCLC Patients

**FIGURE 3:**
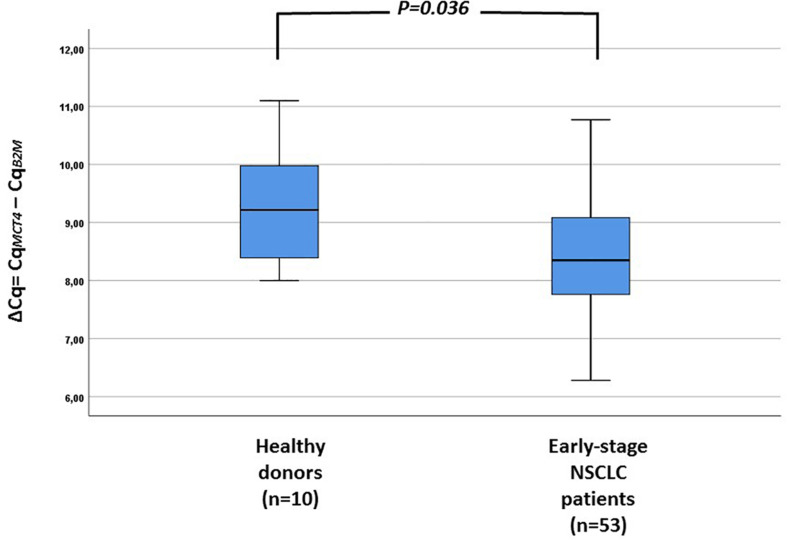
Expression levels of *MCT4* in CTC fractions of HD (*n* = 10) and early stage NSCLC patients (*n* = 53).

**FIGURE 4:**
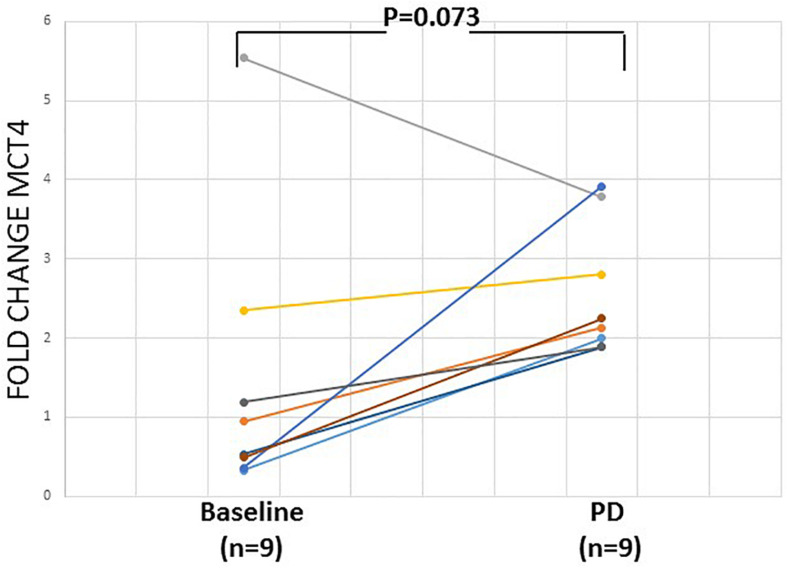
Relative fold change (2^–ΔΔ*Cq*^) of *MCT4 i*n CTCs of 9 pairs of early stage NSCLC patient samples at the baseline and at the time of relapse.

Overexpression of *MCT4* transcripts could be detected in 14/53 (26.4%) patient samples. The evaluation of the differences in the expression of *MCT4* in CTC between HD and early stage NSCLC patients revealed a significantly higher expression of *MCT4.* As can be seen in Figure 3, the overexpression of *MCT4* differed significantly in patient’s samples compared to HD’s samples (*P* = 0.036; Figure 3). For a subgroup of these patients (*n* = 9), peripheral blood samples were available both at baseline and at the time of relapse. In this group, *MCT4* overexpression was observed in 6/18 (33.3%) CTC fraction samples [in 3/9 (33.3%) at baseline and 3/9 (33.3%) at disease progression. In two of these three cases both CTC fractions at baseline and time of relapse were found to be positive for *MCT4* overexpression. There was one case where *MCT4* overexpression was detected only in the baseline, but not at progression disease whereas there was one case where the sample was found to be positive for *MCT4* overexpression in the time of relapse. Moreover, there was only one case where the patient was identified to decrease the expression levels of MCT4 at the time of relapse in respect to the baseline sample but it seemed to remain overexpressed (Figure 4).

### Prognostic Significance of MCT4 Expression in CTCs

**FIGURE 5:**
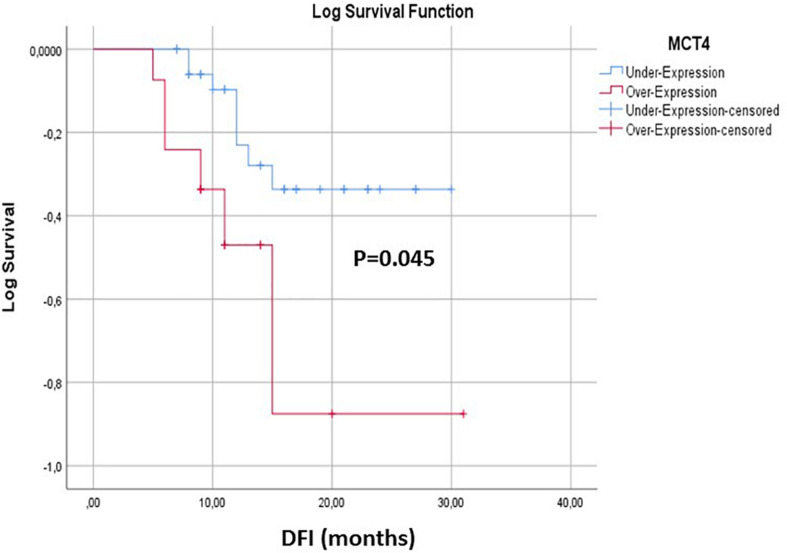
Kaplan–Meier estimates of DFI of early stage NSCLC patients in respect to MCT4 overexpression in CTCs.

The correlation between the expression levels of *MCT4* in CTCs and prognosis was further analyzed. During of follow-up period (median: 12.5, range: 5–31 months) 14/53 (26.4%) NSCLC patients developed metastases. Kaplan–Meier survival analysis demonstrated that patients who overexpressed *MCT4* (*n* = 14) had a significantly shorter DFI than those without *MCT4* overexpression (*P* = 0.045; Figure 5). Moreover, as can been seen in Supplementary Table 2, *MCT4* overexpression was not correlated with gender, smoking history, tumor size, lymph node status, and stage of disease in the population studied (*P* > 0.05).

## Discussion

Increased rates of glycolysis is one of the molecular mechanisms that has been studied for initiation of cancer cell metastasis (Wang et al., 2015). Warburg et al. (1927) have shown that cancer cells have an increased rate of glucose uptake and favored production of lactate, even in the normoxic conditions. It is well-known that cancer cells in order to avoid apoptosis and cellular acidosis export the intracellular lactate and monocarboxylate transporters (MCTs) play a critical role in this exportation (Halestrap and Price, 1999). This mainly performed through the action of *MCT1* and *MCT4* that control intracellular pH in cells relying on high glycolysis rates, such as red blood cells, skeletal muscle cells and tumor cells (Halestrap and Price, 1999; Halestrap, 2013). Although the expression of MCT4 in the tumor microenvironment has been associated with poor prognosis (Nakayama et al., 2012; Baek et al., 2014) and MCT4 has been studied as a new therapeutic target (Kim et al., 2018; Puri and Juvale, 2020). Immunofluoresence has been used for the detection of MCT 1 and MCT4 in cancer patient’s CTC by Kershaw et al. (2015) but so far there is no any other reference on *MCT4* expression at the mRNA level on CTCs.

In the present study, we evaluated for the first time *MCT4* expression in CTC fractions isolated by a size- based EpCAM independent technology (Parsortix) from early stage NSCLC patients using a highly sensitive and specific RT-qPCR assay. *MCT4* plays a critical role in energy production, tumor proliferation and invasion since lactic acid is secreted by cancer cells and acidify the tumor microenvironment (Shu et al., 2016). The inhibition of *MCT4* protein has been suggested as a novel therapeutic approach for many malignancies including NSCLC (Kuo et al., 2020). Bioinformatic analyses of the TCGA datasets demonstrated that *MCT4* is elevated in 9% of lung adenocarcinomas cases. Our findings indicate that *MCT4* is overexpressed at 14/53 (26.4%) on CTC fractions of early stage NSCLC patients. A similar positivity rate (26.4%) was also detected for epithelial markers (*CK18* and *CK19*) while the positivity rate was increased (66%) regarding the EMT marker *VIM* indicating that in NSCLC a large fraction of CTCs is under EMT status which is further supported by the observation that 13 out of 14 (92.8%) of *MCT4* overexpressed samples were found to express at least one EMT marker; conversely, the correspondent proportion of blood samples harboring at least one epithelial marker was 50%. These findings are consistent with previous studies demonstrating that the vast majority of CTC in NSCLC patients express EMT markers (Allard et al., 2004; Lecharpentier et al., 2011; Zhang et al., 2019).

The expression of *MCT4* in the CTC fraction of patients was significantly higher than that observed in the corresponding “PBMC” fraction of HD (*P* = 0.036). Moreover, a higher expression level of *MCT4* in CTC were observed at the time of tumor relapse. This finding, which is in agreement with the reported increased tumoral expression of *MCT4* when the tumor progress to higher grade or metastasis, strongly which suggests that *MCT4* expression is associated with the migration and/or invasion of cancer cells (Gerlinger et al., 2012; Choi et al., 2014; Kim et al., 2015).

According to our results, overexpression of *MCT4* had a prognostic implication for early stage NSCLC patients, since it was associated with significantly reduced DFI; but conversely, there was no correlation with other patients’ clinic-pathological characteristics. On the contrary, neither the expression of epithelial markers nor EMT markers provided prognostic information for NSCLC patients. However, these observations and conclusions have to be been taken with cautious because of the relatively small number of enrolled patients in the study and the low number of observed relapses during the follow-up period and need further confirmation in larger future studies. In a previous study, Ruan et al. (2017) reported that the expression *MCT-4*protein in the primary tumor cells was significantly associated with the depth of tumoral invasion (*P* = 0.034) and with a decreased overall survival (*P* = 0.001).

In recent years, MCT4 inhibitors are still in the discovery phase. Very recently Kuo et al. (2020) suggested a new therapeutic approach for the control of MCT4 in the aerobic glycolysis-preference NSCLC cell subtype. Moreover, the elimination of lactace secretion could be more effective by developing drugs that co-inhibit MCT1 and MCT4. The experimental approach *in vivo* or *in vitro* establishes that not only the inhibition of MCTs can be useful, but also MCTs could serve as vehicles for new anticancer drugs (Baltazar et al., 2014).

## Conclusion

In conclusion, we show that overexpression *MCT4* in CTCs has prognostic significance in early stage NSCLC patients. It would be valuable to extend this study in a prospective large study as well as in different cancers, since most studies so far are based on the expression of *MCT4* in paired fresh frozen tissues. Taking into account the importance of *MCT4* in cancer, its promising potential as a new specific liquid biopsy prognostic biomarker as well as molecular targets for the development of novel cancer therapeutics, we strongly believe that our results will be of importance for both clinical researchers and those who design novel cancer therapeutics.

## Data Availability Statement

The data that support the findings of this study are available from the corresponding author (AM), upon reasonable request.

## Ethics Statement

The studies involving human participants were reviewed and approved by Ethics and Scientific Committee of Thoracic Diseases General Hospital Sotiria. The patients/participants provided their written informed consent to participate in this study.

## Author Contributions

AM conceived the original idea, supervised the findings of this work, and supervised the project. GK and EP contributed to immunostaining and confocal imaging. VG and AK contributed to provision of clinical samples. ET carried out the experiment. AM wrote the manuscript in consultation with EL and VG. EL helped supervise the project. All authors contributed to the article and approved the submitted version.

## Conflict of Interest

The authors declare that the research was conducted in the absence of any commercial or financial relationships that could be construed as a potential conflict of interest.

## Supplementary Material

Supplementary Figure 1 Positive and negative controls of CK/A45/VIM staining. **(A)** Cytospin with H1299 cells stained with CK/A45 (green) anti-mouse, Alexa 488 anti-mouse, VIM (red) anti-rabbit and Alexa 555 anti-rabbit (positive control). **(B)** Cytospin with H1299 cells stained with Alexa 488 anti-mouse, VIM (red) anti-rabbit and Alexa 555 anti-rabbit (negative control for CK). **(C)** Cytospin with H1299 cells stained with CK/A45 (green) anti-mouse, Alexa 488 anti-mouse and Alexa 555 anti-rabbit (negative control for VIM).

Supplementary Figure 2 Phenotypic characterization of CK^*low*^/CK^–^ CTCs. Patients’ samples were stained with DAPI (blue; first column), CK/A45 (green; second column) and VIM (red; third column). The fourth column shows overlays. Representative images from confocal laser scanning microscopy of (A) CK^*low*^/CK^–^/VIM^–^ and (B) CK^*low*^/CK^–^/VIM^+^ CTCs. Scale bars = 10 μm.

Supplementary Table 1 Patients clinicopathological characteristics.

Supplementary Table 2 *MCT4* overexpression in respect to patients clinicopathological characteristics.

Supplementary Table 3 Primers and probe sequence for the RT-qPCR of *MCT4*.

## References

[B1] Allard W. J. Matera J. Miller M. C. Repollet M. Connelly M. C. Rao C. (2004). Tumor cells circulate in the peripheral blood of all major carcinomas but not in healthy subjects or patients with nonmalignant diseases. *Clin. Cancer Res.* 10 6897–6904. 10.1158/1078-0432.CCR-04-0378 15501967

[B2] Baek G. Tse Y. F. Hu Z. Cox D. Buboltz N. McCue P. (2014). MCT4 defines a glycolytic subtype of pancreatic cancer with poor prognosis and unique metabolic dependencies. *Cell Rep.* 9 2233–2249. 10.1016/j.celrep.2014.11.025 25497091

[B3] Baltazar F. Pinheiro C. Morais-Santos F. Azevedo-Silva J. Queirós O. Preto A. (2014). Monocarboxylate transporters as targets and mediators in cancer therapy response. *Histol. Histopathol.* 29 1511–1524. 10.14670/HH-29.1511 24921258

[B4] Choi J. W. Kim Y. Lee J. H. Kim Y. S. (2014). Prognostic significance of lactate/proton symporters MCT1, MCT4, and their chaperone CD147 expressions in urothelial carcinoma of the bladder. *Urology* 84 245.e9–245.e15. 10.1016/j.urology.2014.03.031 24857275

[B5] Chudziak J. Burt D. J. Mohan S. Rothwell D. G. Mesquita B. Antonello J. (2016). Clinical evaluation of a novel microfluidic device for epitope-independent enrichment ofcirculatingtumour cells in patients with small cell lung cancer. *Analyst* 141 669–678. 10.1039/c5an02156a 26605519

[B6] Curry J. M. Tuluc M. Whitaker-Menezes D. Ames J. A. Anantharaman A. Butera A. (2013). Cancer metabolism, stemness and tumor recurrence: MCT1 and MCT4 are functional biomarkers of metabolic symbiosis in head and neck cancer. *Cell Cycle* 12 1371–1384. 10.4161/cc.24092 23574725 PMC3674065

[B7] DeBerardinis R. J. Chandel N. S. (2020). We need to talk about the Warburg effect. *Nat. Metab.* 2 127–129. 10.1038/s42255-020-0172-2 32694689

[B8] Doyen J. Trastour C. Ettore F. Peyrottes I. Toussant N. Gal J. (2014). Expression of the hypoxia-inducible monocarboxylate transporter MCT4 is increased in triple negative breast cancer and correlates independently with clinical outcome. *Biochem. Biophys. Res. Commun.* 451 54–61. 10.1016/j.bbrc.2014.07.050 25058459

[B9] Frick M. A. Feigenberg S. J. Jean-Baptiste S. R. Aguarin L. A. Mendes A. Chinniah C. (2020). Circulating tumor cells are associated with recurrent disease in patients with early-stage non-small cell lung cancer treated with stereotactic body radiotherapy. *Clin. Cancer Res.* 26:2380. 10.1158/1078-0432.CCR-19-2158 PMC9940939 31969332

[B10] Gerlinger M. Santos C. R. Spencer-Dene B. Martinez P. Endesfelder D. Burrell R. A. (2012). Genome-wide RNA interference analysis of renal carcinoma survival regulators identifies MCT4 as a Warburg effect metabolic target. *J. Pathol.* 227 146–156. 10.1002/path.4006 22362593 PMC3504091

[B11] Halestrap A. P. (2013). The SLC16 gene family - structure, role and regulation in health and disease. *Mol. Aspects Med.* 34 337–349. 10.1016/j.mam.2012.05.003 23506875

[B12] Halestrap A. P. Price N. T. (1999). The proton-linked monocarboxylate transporter (MCT) family: structure, function and regulation. *Biochem. J.* 343 (Pt 2), 281–299. 10.1042/0264-6021:3430281 10510291 PMC1220552

[B13] Hanahan D. Weinberg R. A. (2011). Hallmarks of cancer: the next generation. *Cell* 144 646–674. 10.1016/j.cell.2011.02.013 21376230

[B14] Heidrich I. Ačkar L. MossahebiMohammadi P. Pantel K. (2020). Liquid biopsies: potential and challenges. *Int. J. Cancer* 148 528–545. 10.1002/ijc.33217 32683679

[B15] Hvichia G. E. Parveen Z. Wagner C. Janning M. Quidde J. Stein A. (2016). A novel microfluidic platform for size and deformability based separation and the subsequent molecular characterization of viable circulating tumor cells. *Int. J. Cancer* 138 2894–2904. 10.1002/ijc.30007 26789903 PMC5069649

[B16] Kallergi G. Politaki E. Alkahtani S. Stournaras C. Georgoulias V. (2016). Evaluation of isolation methods for circulating tumor cells (CTCs). *Cell Physiol. Biochem.* 40 411–419. 10.1159/000452556 27889762

[B17] Kershaw S. Cummings J. Morris K. Tugwood J. Dive C. (2015). Optimisation of immunofluorescence methods to determine MCT1 and MCT4 expression in circulating tumour cells. *BMC Cancer* 15:387. 10.1186/s12885-015-1382-y 25957999 PMC4436118

[B18] Kim H. K. Lee I. Bang H. Kim H. C. Lee W. Y. Yun S. H. (2018). MCT4 expression is a potential therapeutic target in colorectal cancer with peritoneal carcinomatosis. *Mol. Cancer Ther.* 17 838–848. 10.1158/1535-7163.MCT-17-0535 29483215

[B19] Kim Y. Choi J. W. Lee J. H. Kim Y. S. (2015). Expression of lactate/H? symporters MCT1 and MCT4 and their chaperone CD147 predicts tumor progression in clear cell renal cell carcinoma: immunohistochemical and The Cancer Genome Atlas data analyses. *Hum. Pathol.* 46 104–112. 10.1016/j.humpath.2014.09.013 25456395

[B20] Krebs M. G. Hou J. M. Sloane R. Lancashire L. Priest L. Nonaka D. (2012). Analysis of circulating tumor cells in patients with non-small cell lung cancer using epithelial marker-dependent and -independent approaches. *J. Thorac. Oncol.* 7 306–315. 10.1097/jto.0b013e31823c5c16 22173704

[B21] Kuo T. C. Huang K. Y. Yang S. C. Wu S. Chung W. C. Chang Y. L. (2020). Monocarboxylate transporter 4 is a therapeutic target in non-small cell lung cancer with aerobic glycolysis preference. *Mol. Ther. Oncolytics* 18 189–201. 10.1016/j.omto.2020.06.012 32695876 PMC7364124

[B22] Lecharpentier A. Vielh P. Perez-Moreno P. Planchard D. Soria J. C. Farace F. (2011). Detection of circulating tumour cells with a hybrid (epithelial/mesenchymal) phenotype in patients with metastatic non-small cell lung cancer. *Br. J. Cancer* 105 1338–1341. 10.1038/bjc.2011.405 21970878 PMC3241564

[B23] Lianidou E. S. Markou A. Strati A. (2015). The role of CTCs as tumor biomarkers. *Adv. Exp. Med. Biol.* 867 341–367. 10.1007/978-94-017-7215-0_21 26530376

[B24] Livak K. J. Schmittgen T. D. (2001). Analysis of relative gene expression data using real-time quantitative PCR and the 2(-Delta DeltaC(T)) method. *Methods* 25 402–408. 10.1006/meth.2001.1262 11846609

[B25] Luo W. Rao M. Qu J. Luo D. (2018). Applications of liquid biopsy in lung cancer-diagnosis, prognosis prediction, and disease monitoring. *Am. J. Transl. Res.* 10 3911–3923. 30662639 PMC6325529

[B26] Markou A. Lazaridou M. Paraskevopoulos P. Chen S. Swierczewska M. Budna J. (2018). Multiplex gene expression profiling of in vivo isolated circulating tumor cells in high-risk prostate cancer patients. *Clin. Chem.* 64 297–306. 10.1373/clinchem.2017.275503 29122836

[B27] Meng S. Tripathy D. Frenkel E. P. Shete S. Naftalis E. Z. Huth J. F. (2004). Circulating tumor cells in patients with breast cancer dormancy. *Clin. Cancer Res.* 10 8152–8162. 15623589 10.1158/1078-0432.CCR-04-1110

[B28] Meredith D. Christian H. C. (2008). The SLC16 monocaboxylate transporter family. *Xenobiotica* 38 1072–1106. 10.1080/00498250802010868 18668440

[B29] Molina J. R. Yang P. Cassivi S. D. Schild S. E. Adjei A. A. (2008). Non-small cell lung cancer: epidemiology, risk factors, treatment, and survivorship. *Mayo Clin. Proc.* 83 584–594. 10.4065/83.5.584 18452692 PMC2718421

[B30] Nakayama Y. Torigoe T. Inoue Y. Minagawa N. Izumi H. Kohno K. (2012). Prognostic significance of monocarboxylate transporter 4 expression in patients with colorectal cancer. *Exp. Ther. Med.* 3 25–30. 10.3892/etm.2011.361 22969839 PMC3438655

[B31] Pawlikowska P. Faugeroux V. Oulhen M. Aberlenc A. Tayoun T. Pailler E. (2019). Circulating tumor cells (CTCs) for the noninvasive monitoring and personalization of non-small cell lung cancer (NSCLC) therapies. *J. Thorac. Dis.* 11 S45–S56. 30775027 10.21037/jtd.2018.12.80 PMC6353742

[B32] Poole R. C. Halestrap A. P. (1993). Transport of lactate and other monocarboxylates across mammalian plasma membranes. *Am. J. Physiol.* 264 C761–C782. 10.1152/ajpcell.1993.264.4.C761 8476015

[B33] Porras T. B. Kaur P. Ring A. Schechter N. Lang J. E. (2018). Challenges in using liquid biopsies for gene expression profiling. *Oncotarget* 9 7036–7053. 10.18632/oncotarget.24140 29467948 PMC5805534

[B34] Puri S. Juvale K. (2020). Monocarboxylate transporter 1 and 4 inhibitors as potential therapeutics for treating solid tumours: a review with structure-activity relationship insights. *Eur. J. Med. Chem.* 199:112393. 10.1016/j.ejmech.2020.112393 32388280

[B35] Ruan Y. Zeng F. Cheng Z. Zhao X. Fu P. Chen H. (2017). High expression of monocarboxylate transporter 4 predicts poor prognosis in patients with lung adenocarcinoma. *Oncol. Lett.* 14 5727–5734. 10.3892/ol.2017.6964 29113201 PMC5661367

[B36] Shu Q. H. Ge Y. S. Ma H. X. Gao X. Q. Pan J. Liu D. (2016). Prognostic value of polarized macrophages in patients with hepatocellular carcinoma after curative resection. *J. Mol. Med.* 94 155–171. 26843477 10.1111/jcmm.12787 PMC4882981

[B37] Siegel R. L. Miller K. D. Jemal A. (2015). Cancer statistics, 2015. *CA Cancer J. Clin.* 65 5–29. 10.3322/caac.21254 25559415

[B38] Strati A. Koutsodontis G. Papaxoinis G. Angelidis I. Zavridou M. Economopoulou P. (2017). Prognostic significance of PD-L1 expression on circulating tumor cells in patients with head and neck squamous cell carcinoma. *Ann. Oncol.* 28 1923–1933. 10.1093/annonc/mdx206 28838214

[B39] Strati A. Nikolaou M. Georgoulias V. Lianidou E. S. (2019). Prognostic significance of TWIST1, CD24, CD44, and ALDH1 transcript quantification in EpCAM-positive circulating tumor cells from early stage breast cancer patients. *Cells* 8:652. 10.3390/cells8070652 31261917 PMC6679222

[B40] Tamminga M. de Wit S. Schuuring E. Timens W. Terstappen L. W. M. M. Hiltermann T. J. N. (2019). Circulating tumor cells in lung cancer are prognostic and predictive for worse tumor response in both targeted- and chemotherapy. *Transl. Lung Cancer Res.* 8 854–861. 10.21037/tlcr.2019.11.06 32010564 PMC6976367

[B41] Wang R. Lu Y. Y. Fan D. M. (2015). Reasons for cancer metastasis: a holistic perspective. *Mol. Clin. Oncol.* 3 1199–1202. 10.3892/mco.2015.623 26807220 PMC4665943

[B42] Wang W. Song Z. Zhang Y. A. (2017). Comparison of ddPCR and ARMS for detecting EGFR T790M status in ctDNA from advanced NSCLC patients with acquired EGFR-TKI resistance. *Cancer Med.* 6 154–162. 10.1002/cam4.978 28000387 PMC5269560

[B43] Warburg O. (1956). On the origin of cancer cells. *Science* 123 309–314. 10.1126/science.123.3191.309 13298683

[B44] Warburg O. Wind F. Negelstein E. (1927). The metabolism of tumors in the body. *J. Gen. Physiol.* 8 519–530. 10.1085/jgp.8.6.519 19872213 PMC2140820

[B45] Wu Y. L. Tsuboi M. He J. John T. Grohe C. Majem M. (2020). Osimertinib in resected *EGFR*-mutated non-small-cell lung cancer. *N. Engl. J. Med.* 383 1711–1723. 10.1056/NEJMoa2027071 32955177

[B46] Zavridou M. Mastoraki S. Strati A. Tzanikou E. Chimonidou M. Lianidou E. (2018). Evaluation of preanalytical conditions and implementation of quality control steps for reliable gene expression and DNA methylation analyses in liquid biopsies. *Clin. Chem.* 64 1522–1533. 10.1373/clinchem.2018.292318 30018056

[B47] Zhang X. Chang N. Yang G. Zhang Y. Ye M. Cao J. (2017). A comparison of ARMS-Plus and droplet digital PCR for detecting EGFR activating mutations in plasma. *Oncotarget* 8 112014–112023. 10.18632/oncotarget.22997 29340107 PMC5762375

[B48] Zhang X. Wei L. Li J. Zheng J. Zhang S. Zhou J. (2019). Epithelial-mesenchymal transition phenotype of circulating tumor cells is associated with distant metastasis in patients with NSCLC. *Mol. Med. Rep.* 19 601–608. 30483792 10.3892/mmr.2018.9684

[B49] Zhu J. Wu Y. N. Zhang W. Zhang X. M. Ding X. Li H. Q. (2014). Monocarboxylate transporter 4 facilitates cell proliferation and migration and is associated with poor prognosis in oral squamous cell carcinoma patients. *PLoS One* 9:e87904. 10.1371/journal.pone.0087904 24498219 PMC3907573

