# Supplementary figures and images for: Evaluation of Monocarboxylate Transporter 4 (MCT4) Expression and Its Prognostic Significance in Circulating Tumor Cells From Patients With Early Stage Non-Small-Cell Lung Cancer
Source: Front Cell Dev Biol. 2021 Apr 22;9:641978. doi: 10.3389/fcell.2021.641978 (PMC8100022; doi:10.3389/fcell.2021.641978)

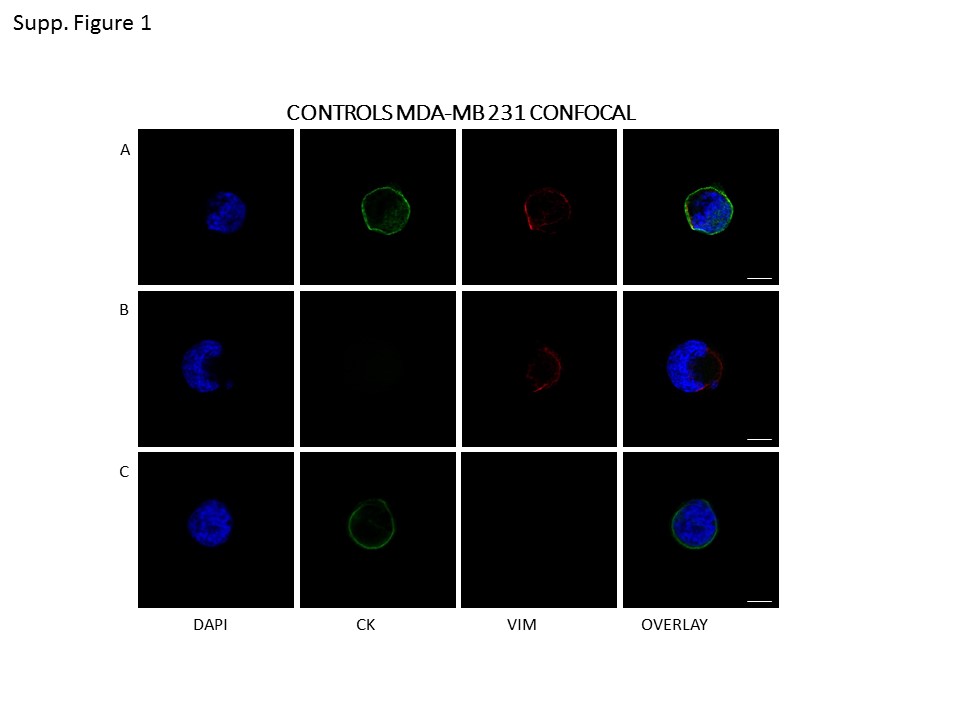

Supplement: Supplementary Figure 1 — Positive and negative controls of CK/A45/VIM staining. (A) Cytospin with H1299 cells stained with CK/A45 (green) anti-mouse, Alexa 488 anti-mouse, VIM (red) anti-rabbit and Alexa 555 anti-rabbit (positive control). (B) Cytospin with H1299 cells stained with Alexa 488 anti-mouse, VIM (red) anti-rabbit and Alexa 555 anti-rabbit (negative control for CK). (C) Cytospin with H1299 cells stained with CK/A45 (green) anti-mouse, Alexa 488 anti-mouse and Alexa 555 anti-rabbit (negative control for VIM). [file Image_1.jpg]

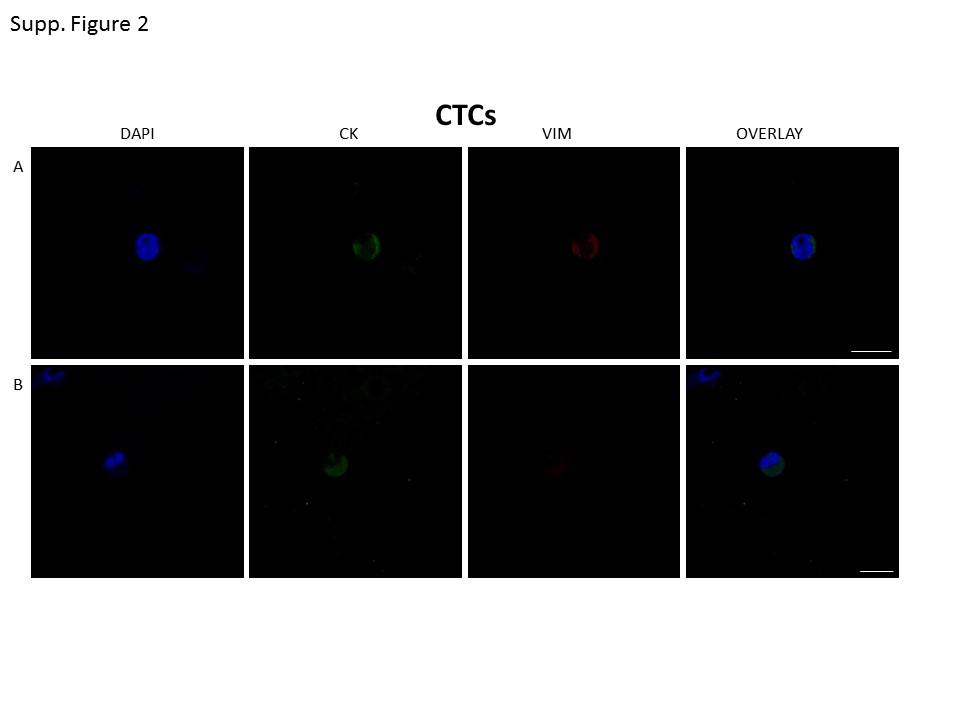

Supplement: Supplementary Figure 2 — Phenotypic characterization of CKlow/CK– CTCs. Patients’ samples were stained with DAPI (blue; first column), CK/A45 (green; second column) and VIM (red; third column). The fourth column shows overlays. Representative images from confocal laser scanning microscopy of (A) CKlow/CK–/VIM– and (B) CKlow/CK–/VIM+ CTCs. Scale bars = 10 μm. [file Image_2.jpg]
