# Supplementary material for: Evaluation of Monocarboxylate Transporter 4 (MCT4) Expression and Its Prognostic Significance in Circulating Tumor Cells From Patients With Early Stage Non-Small-Cell Lung Cancer
Source: Front Cell Dev Biol. 2021 Apr 22;9:641978. doi: 10.3389/fcell.2021.641978 (PMC8100022; doi:10.3389/fcell.2021.641978)
Supplement: Supplementary Table 1 — Patients clinicopathological characteristics. [file Table_1.DOCX]

| **Average age (range)** | 65.2 (39-81) |
| --- | --- |
| **Gender** | |
| Male | 32 |
| Female | 21 |
| **Smoking History** | |
| Current | 26 |
| Former | 18 |
| Never | 3 |
| **Histology** | |
| Adenocarcinoma | 23 |
| Squamous Cell Carcinoma | 26 |
| Other | 4 |
| **Pathological Stage of lung cancer** |  |
| I | 29 |
| II | 9 |
| IIIA | 12 |
| **Tumor Size** |  |
| ≤2 cm | 8 |
| >2-3 cm | 14 |
| >3-5 cm | 17 |
| >5-7 cm | 6 |
| >7 cm | 8 |
| **Lymph nodes** |  |
| 0 | 38 |
| ≥1 | 15 |
