# Supplementary material for: Evaluation of Monocarboxylate Transporter 4 (MCT4) Expression and Its Prognostic Significance in Circulating Tumor Cells From Patients With Early Stage Non-Small-Cell Lung Cancer
Source: Front Cell Dev Biol. 2021 Apr 22;9:641978. doi: 10.3389/fcell.2021.641978 (PMC8100022; doi:10.3389/fcell.2021.641978)
Supplement: Supplementary Table 2 — MCT4 overexpression in respect to patients clinicopathological characteristics. [file Table_2.doc]

**Suppl. Table 2. *MCT4* overexpression in respect to patients clinicopathological characteristics**

|  | **All Patients**  **No %** | **MCT4 Overexpression**  **No % Pa** |
| --- | --- | --- |
| **Patients** | 53 | 14 26.4 |
| **Gender**  **Male**  **Female** | 32 60.4  21 39.6 | 9 28.1 0.730  5 23.8 |
| **Histology Grade**  **Adenocarcinoma Squamous Cell Carcinoma**  **Other** | 23 43.4  26 49.0  4 7.5 | 3 13.0 0.136  10 28.5  1 25 |
| **Tumor Size**  **≤2cm**  **>2-3cm**  **>3-5cm**  **>5-7cm**  **>7cm** | 8 15.1  14 26.4  17 32.0  6 11.3  8 15.1 | 0 0 0.278  3 21.4  7 41.2  1 16.7  3 37.5 |
| **Lymph Node**  **0**  **≥1** | 38 71.7  15 28.3 | 10 26.3 0.979  4 26.7 |
| **Smoking History**  **Never**  **Current**  **Former** | 3 5.7  17 32.1  14 26.4 | 0 0 0.797  9 52.9  4 28.6 |
