# Supplementary material for: Evaluation of Monocarboxylate Transporter 4 (MCT4) Expression and Its Prognostic Significance in Circulating Tumor Cells From Patients With Early Stage Non-Small-Cell Lung Cancer
Source: Front Cell Dev Biol. 2021 Apr 22;9:641978. doi: 10.3389/fcell.2021.641978 (PMC8100022; doi:10.3389/fcell.2021.641978)
Supplement: Supplementary Table 3 — Primers and probe sequence for the RT-qPCR of MCT4. [file Table_3.doc]

**Suppl.Table 3:** Primers and probe sequence for the RT-qPCR of MCT4

|  |  |
| --- | --- |
| FORWARD | 5’-CCATGCTCTACGGGACAGGT-3’ |
| REVERSE | 5’-TCACGGGGTTGGGTTTGG-3’ |
| Probe | 5’-FAM-TGTGCGTGAACCGCTTTGGCTG-BHQ-3’ |
